# Epigenetics of pregnancy: looking beyond the DNA code

**DOI:** 10.1007/s10815-022-02451-x

**Published:** 2022-03-17

**Authors:** Daniela Zuccarello, Ugo Sorrentino, Valeria Brasson, Loris Marin, Chiara Piccolo, Antonio Capalbo, Alessandra Andrisani, Matteo Cassina

**Affiliations:** 1 Clinical Genetics Unit, Department of Women’s and Children’s Health, University Hospital of Padova, Padua, Italy; 2 Gynaecological Clinic, Department of Women’s and Children’s Health, University of Padua, Padua, Italy; 3 Igenomix Italy, Marostica, VI Italy

**Keywords:** Epigenetics, miRNA, Pregnancy, Review, Embryo and fetus development, Brain development

## Abstract

Epigenetics is the branch of genetics that studies the different mechanisms that influence gene expression without direct modification of the DNA sequence. An ever-increasing amount of evidence suggests that such regulatory processes may play a pivotal role both in the initiation of pregnancy and in the later processes of embryonic and fetal development, thus determining long-term effects even in adult life. In this narrative review, we summarize the current knowledge on the role of epigenetics in pregnancy, from its most studied and well-known mechanisms to the new frontiers of epigenetic regulation, such as the role of ncRNAs and the effects of the gestational environment on fetal brain development. Epigenetic mechanisms in pregnancy are a dynamic phenomenon that responds both to maternal–fetal and environmental factors, which can influence and modify the embryo-fetal development during the various gestational phases. Therefore, we also recapitulate the effects of the most notable environmental factors that can affect pregnancy and prenatal development, such as maternal nutrition, stress hormones, microbiome, and teratogens, focusing on their ability to cause epigenetic modifications in the gestational environment and ultimately in the fetus. Despite the promising advancements in the knowledge of epigenetics in pregnancy, more experience and data on this topic are still needed. A better understanding of epigenetic regulation in pregnancy could in fact prove valuable towards a better management of both physiological pregnancies and assisted reproduction treatments, other than allowing to better comprehend the origin of multifactorial pathological conditions such as neurodevelopmental disorders.

## Introduction

The British epidemiologist David Barker first introduced the concept that “the womb may be more important than the home”, emphasizing the role of the gestational environment as a regulatory staple in the development of the embryo, of the fetus and, ultimately, of the adult [1].

After fertilization, oviductal and endometrial fluids nurture the embryo and regulate its development, before being replaced in this role by the placenta in the further stages of the pregnancy, while the embryo, and later the fetus, interact with the maternal environment in order to further its own implantation and development. This embryo-maternal and fetus-maternal crosstalk [2–6] is finely regulated by numerous cellular pathways, including epigenetic mechanisms.

Epigenetics is defined as the study of heritable changes in gene expression that do not involve modifications to the underlying DNA sequence. Knowledge of the phenomena related to such regulatory mechanisms carries a huge relevance in both physiology and pathophysiology. Both parental and environmental factors (such as nutrition, stress, socioeconomic status and exposure to teratogens and other environmental drivers) have been demonstrated to modulate prenatal development both in the preconception phase and afterwards during the pregnancy, via changes to the DNA methylation patterns (also known as methylome), histone modifications, and/or non-coding RNA (ncRNA) system.

Extensive evidence has progressively emerged that the gestational environment determines a remarkable impact on an epigenetic level, at least with two different mechanisms: by directly regulating the stages of implantation and placentation; and widely remodeling epigenetic patterns during prenatal development, thus determining long-term outcomes in the offspring [4, 5, 7–10].

This narrative review aims to recapitulate both the already established and the newly emerging environmental and parental factors that regulate pregnancy through epigenetic mechanisms.

## Main epigenetics mechanisms

The epigenetic modulation of gene expression begins in the gametes and zygote through genomic imprinting and then continues throughout the whole gestation with changes to the fetal and maternal methyloma and the specific action of the ncRNA system. The main processes involved in the epigenetic regulation of pregnancy, namely the DNA methylation, the histone post-translational modifications, the non-coding RNA and the genomic imprinting, are summarized in Table 1.

**Table 1:** Main epigenetic processes involved in the regulation of pregnancy

Processes	Mechanisms	Enzymes/molecules	Effect
DNA methylation	Methylation of CpG and CGIs	-DNMT1 -DNMT3A -DNMT3B -TET family -MBD4 -TDG	-Transcription silencing (sporadically transcription permissive) -Chromatin remodeling
Histone post-translational modification	Histones’: Acetylation (A) Methylation (M) Phosphorylation (P) Ubiquitination (U)	-HATs (6 groups) -HDACs (4 classes, 18 enzymes) -HMTs -HDMs -DUBs (2 classes)	-A: ↑ gene expression -M: 1. regulation of imprinting 2. ↓or ↑ gene expression -P: 1. DNA damage repair 2. Chromatin compaction 3. Transcription modulation -U: 1. DNA damage signalling 2. Protein translocation 3. Transcription regulation 4. Cellular signaling modulation
ncRNAs system	-RNA–DNA interaction -RNA-RNA interaction -RNA–protein interaction -Protein recruitment (scaffolding &/or sponging)	1. sncRNAs: -siRNAs -miRNAs -piRNAs 2. lncRNAs: -lincRNAs -ilncRNAs -eRNAs (including promoter/UTR/ telomere-associated lncRNAs)	-Gene expression regulation -Promoter silencing/enhancing -RNA interference -mRNA splicing regulation -Histone-modifying complexes driver -Methylation processes involving -Post-transcriptional regulation
Imprinting	Methylation of: -X-Chromosome -DMRs/ICRs -Histones	DNMT3A DNMT3B DNMT3L	-X- Inactivation -Parental/genomic imprinting control -Imprinting maintenance

*DNMT*, DNA methyltransferase; *TET*, ten-eleven translocation methylcytosine dioxygenases; *MBD4*, methyl-CpG binding protein 4; *TDG*, thymine DNAglycosylase; (*A*), acetylation; (*M*), methylation; (*P*), phosphorylation; (*U*), ubiquitination; *HATs*, histone acetyltransferases; *HDACs*, histone deacetylases; *HMTs*, histone methyltransferases; *HDMs*, histone demethylases; *DUBs*, deubiquitinating enzymes; *ncRNAs*, non-coding RNAs; *sncRNAs*, short ncRNAs; *siRNAs*, short interfering RNAs; *miRNAs*, microRNAs; *piRNAs*, piwi interacting RNAs; *lncRNAs*, long ncRNAs; *lincRNAs*, long intergenic ncRNAs; *ilncRNAs*, intronic long ncRNAs; *eRNAs*, enhancer long ncRNAs; *DMRs*, differentially methylated regions; *ICRs*, imprinting control regions

### DNA methylation

DNA methylation is the most widespread epigenetic modification of the human genome. The addition of a methyl group to the fifth carbon of the pyrimidine cytosine ring determines long-term silencing in processes such as genomic imprinting, tissue-specific regulation of gene expression, X-chromosome inactivation, and silencing of repetitive DNA elements. Correct patterns of DNA methylation, both prenatally and postnatally, are required for normal human development [11–14].

In multicellular eukaryotes, DNA methylation mostly occurs at cytosines of CpG (C–phosphate–G) dinucleotides; highly methylated sequences can also be found in satellite DNAs, repetitive element and non-repetitive intergenic DNA. When established on promoters or enhancers, methylation can repress transcription either directly, by inhibition of transcription factor binding, or indirectly, through recruitment of methyl-binding proteins and chromatin modifiers [11–15]. Even though the most widespread and well-known effect of DNA methylation is long-term silencing of transcription, it should be noted that recent works suggested that in some instances, hypermethylated promoters and enhancers could be permissive to transcription instead [16].

While most of the CpG sites in human DNA are methylated, the CpG-rich regions named CpG islands, that are located in approximately 60% of the promoters of human genes, display a baseline unmethylated pattern. Methylation of CpG islands located in the promoter region of a gene usually inhibits the transcription of that gene due to binding of methyl-CpG binding proteins, as they recruit chromatin remodelers, histone deacetylases, and methylases to the promoter of the gene, thereby blocking transcription [17, 18].

### Histone post-translational modifications

Gene expression is also influenced by a variety of post-translational modifications to histones. Acetylating, methylating, phosphorylating, and ubiquitinating enzymes act alone or in combination to control chromatin compaction [19–22]. Histone acetylation generally results in higher gene expression, with rare exceptions [23–25]. Histone methylation has both permissive or repressive effect on transcription, depending on the location of target amino acid residues in the histone tail or on the number of methyl groups attached [9, 26, 27]. Histone phosphorylation is regulated by kinases (that bind phosphate groups) and phosphatases (which detach the phosphates) and has at least three activities: DNA damage repair, control of chromatin compaction connected with mitosis and meiosis, and modulation of transcriptional function [28, 29]. Histone mono-ubiquitination has a crucial role in protein translocation, DNA damage signaling, and regulation of transcription. On the other hand, poly-ubiquitination flags proteins as a mark for degradation or activation in some signaling pathways [22, 30].

Several studies and evidences report that precise chromatin modification patterns occur in the fetal membrane and decidua cells, changing dynamically during normal and pathological pregnancies [31].

### Non-coding RNAs system

While it is known that most human transcripts are not translated, many of them actively participate in essential cellular functions nonetheless. Specifically, ncRNAs are a family of RNAs that do not encode functional proteins but are involved in pivotal regulatory and housekeeping mechanisms [32, 33].

The regulatory ncRNAs are divided into two main categories, based on size: short-chain non-coding RNAs (sncRNAs, including siRNAs, miRNAs, and piRNAs) when shorter than 200 nucleotides; long-chain non-coding RNA (lncRNAs, including lincRNAs, ilncRNAs, and eRNAs) when longer than 200 nucleotides. ncRNAs were originally thought to be only involved in gene expression at the post-transcriptional level, while nowadays it has been largely established that non-coding RNAs function as the most common regulatory RNAs, both at pre and post transcriptional level (e.g., by promoter silencing or RNA interference mechanisms), and are also involved in epigenetic control. Many of these transcripts are in fact necessary for proper targeting of histone modifying complexes or participate in the DNA methylation process and genomic imprinting. For such reasons, it has been proposed that a contemporary definition of epigenetics should also include the gene silencing or upregulation mediated by ncRNAs [34–36].

In particular, the study of miRNAs expression and transport appears to be the next frontier in terms of improving the understanding of epigenetic mechanisms in fetal development and fetal-maternal crosstalk. Their role in broader effects on fetal and long-term human development is yet to be fully investigated and could represent a promising research field for future studies, which could even expand to the optimization of in vitro fertilization [37, 38].

### Imprinting

During gametogenesis and then again in the early moments after fertilization, mammals undergo a two-step DNA methylation reprogramming that affects more than 80% of the genome. Two events of extensive erasure occur in the primordial germ cells and pre-implantation embryo, followed by de novo DNA methylation, with differential kinetics and patterns during male and female gametogenesis and within cell lineage specification in post-implantation development. [37–41]. Additionally, the process of X-chromosome inactivation will occur in female embryos, leaving only one copy of almost all of the X-linked genes to be expressed [39].

However, specific differentially methylated regions (DMRs), in which DNA is methylated on one specific parental allele, escape the reprogramming process that happens in the pre-implantation embryo [37]. This phenomenon, that allows parent-of–origin specific gene expression, is defined as genomic imprinting. Most imprinted genes are gathered in distinct clusters of the size of about 1 Mb and contain both maternally and paternally expressed genes. In addition to protein-coding genes, these clusters typically contain lncRNA, which can regulate the imprinting of the nearby genes. Regulation of the clustered genes is coordinated through short DNA sequences called imprinting control regions (ICRs). Also, recent evidence is starting to highlight the contribution of post-translational histone modifications to the regulation of imprinting [42, 43].

The disruption of genomic imprinting leads to largely established human diseases with recognizable clinical features such as Beckwith-Wiedemann syndrome (MIM 130,650), Silver-Russell syndrome (MIM 180,860), Prader-Willi syndrome (MIM 176,270), and Angelman syndrome (MIM 105,830), but less dramatic and more subtle changes in the imprinted patterns, especially concerning multi-locus imprinting disturbances (MLID), can instead modulate fetal growth, resource acquisition, and organogenesis [44–46].

**Fig. 1:**
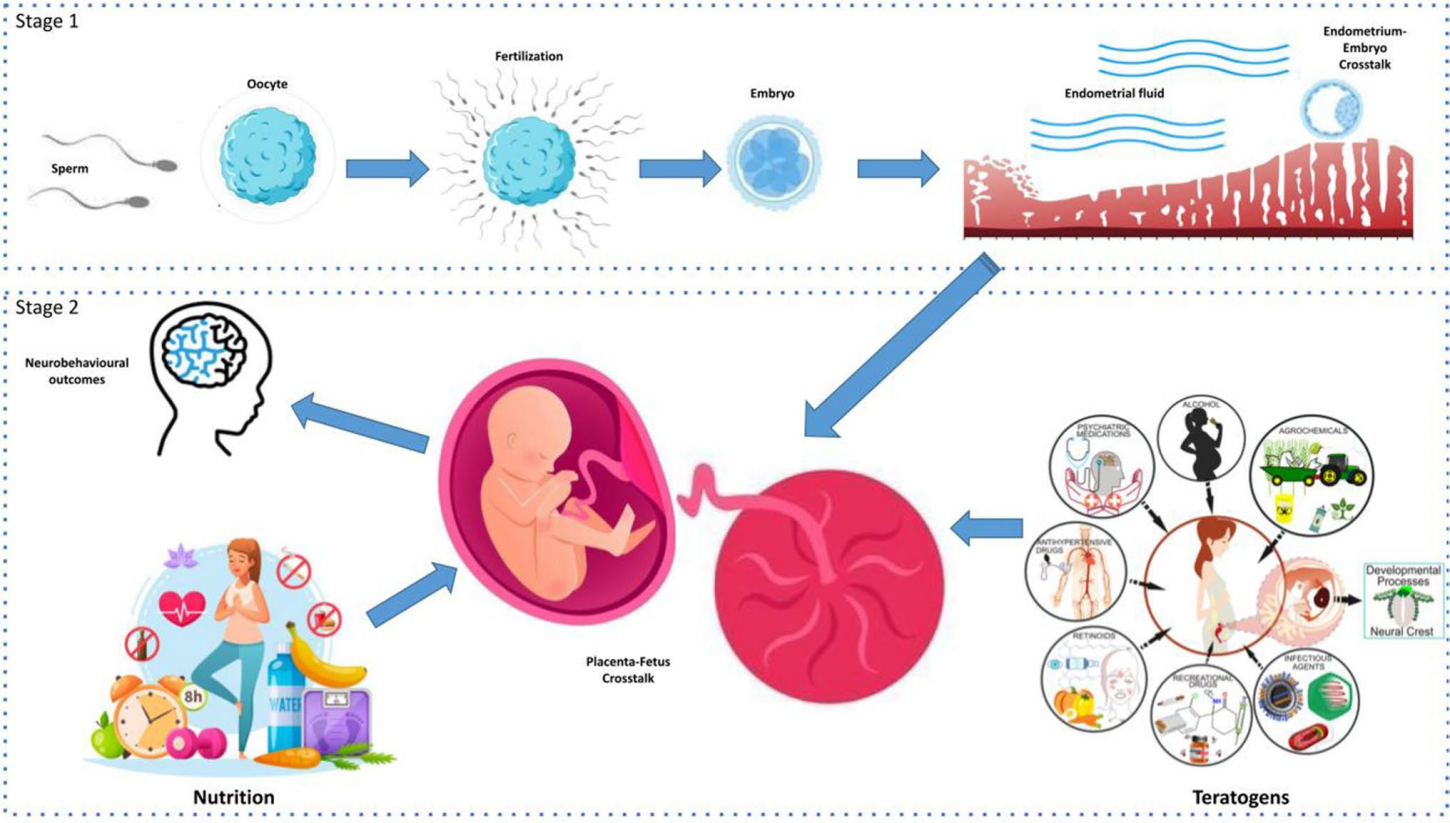
Hot spots of epigenetic action. Stage 1: from gametes to embryo-endometrium cross-talk; Stage 2: from placenta-fetus cross-talk to brain development, with an overview on environmental factors as well (nutrition and teratogens). Modified from Cerrizuela S et al, Birth Defects Res. 2020; other pictures obtained by Vecteezy.com

There are many epigenetic action’s hot spots along the entire pregnancy process, and it would have been too long and purely didactic to analyze them all in detail. We have therefore chosen the most significant steps and those for which there is already literature data that allow a reasoned review of the causative mechanism. Figure 1 details the main hot spots, divided into stage 1 (from gametes to embryo-endometrium cross-talk) and stage 2 (from placenta-fetus cross-talk to brain development, with an overview on environmental factors as well).

## Hot spots of epigenetic action in gametes and embryo stage

### Oocytes and spermatozoa

In addition to determining parent-of-origin specific gene expression by genomic imprinting, the epigenetic reprogramming that happens during gametogenesis regulates gametes development, and its disruption can also reverberate in the zygote, embryo, and postnatal life.

Fertile sperm production requires effective epigenetic regulation. In male gametes, epigenetic patterns are established soon after the demethylation event has occurred in the primordial germ cells. The sperm epigenome is well specialized because of its particular requirements in terms of motility and protection against the female reproductive tract’s hostile environment, exhibiting a unique pattern of histone modifications that includes both activating and silencing marks in the promoters of genes associated with development [47, 48]; sperm chromatin is highly compacted and organized, and spermatogenesis has been associated with fluctuating level of ncRNAs, particularly endo-siRNAs [49].

Sperm epigenetic abnormalities are associated with infertility, reduced embryogenesis capability, and alterations in early embryo development [50, 51], and the study of sperm epigenome is being evaluated as a potential diagnostic tool for idiopathic recurrent pregnancy loss and infertility [47, 50–55].

In the oocytes, de novo DNA methylation does not start as immediately as in their male counterparts and appears to be largely dispensable for the early oocyte development [56]. DNA methylation establishment in the oocyte is dynamically controlled during oocyte growth and it is proximally regulated by transcription events. As a result, DNA methylation is mainly restricted to the transcribed gene bodies, while intergenic regions are hypomethylated [57–59], as non-CpG hypermethylation plays an active role in gene expression regulation during oocyte maturation, potentially through the incorporation with transcription factors [57]. Any adverse factor altering the normal transcription program could therefore perturb the correct methylation patterns [60, 61]. Numerous studies also managed to identify maternal effect genes (MEGs) which encode factors that are present in the oocyte and are required for embryonic development. Pathogenic variants in MEGs or the absence of MEG factors are correlated to adverse outcomes, like zygotic cleavage failure, imprinting disorders, and structural birth defects [62–64]. There is also growing evidence that variants in the maternal genome affecting the imprinting status of the oocyte could cause MLID [44].

### Fertilization and early embryo development

Fertilization is defined as the event when terminally differentiated gametes combine into a totipotent zygote, ultimately giving rise to the embryo. The earliest stages of embryonic development are characterized by major epigenetic remodeling events that include parental DNA methylation erasure and reprogramming (with the exception of DMRs), chromatin folding establishment, and spatial reorganization of the genome [65–67]. The precision and the timing of such processes are pivotal for avoiding developmental defects or embryonic lethality, as an incorrect establishment of the embryonic epigenome would cause a defective zygotic genome activation (ZGA). Such a brief but crucial phase represents a window of vulnerability towards interfering maternal and environmental factors (such as chronic metabolic disorders, polycystic ovary syndrome, diet, and teratogen exposure), as highlighted by an ever-increasing number of studies [68–71]. In particular, it has been suggested that the association between assisted reproductive treatment (ART) procedures and slightly higher frequences of imprinting syndromes may be due to ART causing an abnormal epigenome in the offspring [71]. Also, animal models suggested that chromatin changes occurring in the pluripotent embryo could determine regulatory information whose effects would reverberate in the adult life in terms of growth and brain development [72]. A better comprehension of the underlying mechanisms and their consequences could therefore improve diagnosis and treatment of human infertility and diseases.

### Pre-receptive and receptive endometrium

Embryonal implantation is a crucial step of pregnancy establishment in mammals. The sensitivity of the endometrium to implanting embryos is commonly categorized into pre-receptive, receptive, and refractory phases. This period of receptivity, called “window of implantation” [73], is short and results from the programmed sequence of the action of estrogen and progesterone on the endometrium via multiple paracrine, juxtacrine, and autocrine signaling [26, 74].

High levels of progesterone in the post-ovulatory phase modulate the endometrial gene expression required for implantation by regulating DNA methylation [75–77], and alterations in the DNA methylation of 448 sites have been associated with defective endometrial receptivity and consequently in recurrent pregnancy failure [75, 78]. Moreover, different non-coding transcripts like lncRNAs and miRNAs are involved in endometrial receptivity regulation [79].

Several association studies have evaluated miRNA expression levels in the receptive and pre-receptive endometrium in fertile women [80–83]. The miR-30 family members (primarily miR-30b and miR-30d) were demonstrated to be significantly upregulated [84], while miR-494 and miR-923 were downregulated during the receptive phase. miR-30b and miR-30d were also consistently found to be elevated in the mid-secretory as compared with the proliferative phase. Expression arrays demonstrated that embryos treated with miR-30d exhibited increased expression of ten genes, including those encoding adhesion molecules such as ITGB3 (Integrin Subunit Beta 3), ITGA7 (Integrin Subunit Alpha 7), and CDH5 (Cadherin 5) [85].

A significant inverse association has been found between miR-31 and both FOXP3, a transcription factor for T regulatory cells, and CXCL12, a chemoattractant for uterine natural killer cells, which participates in the creation of an immune-tolerant environment in the secretory phase [86]. The modulation of immune response is considered an important target for miRNAs, because major histocompatibility complexes such as HLA-G appear to be extensively regulated by noncoding RNAs such as miR-133a [87, 88].

Also, members of the miR-17–92 cluster (including miR-17, miR-18a, miR-19a, miR-20a, miR-19b-1, and miR-92a-1) are upregulated at the implantation sites of the receptive uterus in humans. The functions of endometrial miR-17–92 during implantation are unclear. The cluster is known to target TGF-b signalling, which modulates events in decidualization and implantation [89].

Moreover, the miRNA-200 family (miR-200a, miR-200b, miR200c, miR-141, and miR-429) showed a differential expression in the receptive phase as well, when they appear to be downregulated [89].

Finally, by suppressing Dicer, miRNAs from the Lethal-7 family can regulate the expression of other miRNAs. It has been suggested that high levels of let-7 in the receptive uterus could be important for differentiation of a receptive endometrium [87].

Interestingly, histone acetylation is also involved in the early endometrial processes, being implicated in the vascular endothelial growth factor pathway during angiogenesis. Studies using histone deacetylase inhibitors suggest an involvement in endometrial proliferation and differentiation as well [77].

### Decidualization

The term decidualization defines the functional and morphological transition of the endometrial cells to form the cellular environment into which the blastocyst is able to implant itself. Defects in decidualization can lead to recurrent implantation failure and recurrent spontaneous abortion [90, 91]. Human endometrial stem cells (hESC) have been decidualized in vitro to explore the effects of decidualization on miRNA expression. Studies found that miRNA expression profiles of decidualized hESC and control hESC departed from each other. According to Tochigi et al. [92], hESC transfection of miR-542-3p suppressed IGFBP-1 (insulin-like growth factor-binding protein) expression, leading to *PRL* (prolactin) and WNT4 suppression and thus to the inhibition of decidualization in human endometrial stroma cells. This suggests an important role of miR-542-3p in regulation of endometrial decidualization. Estrella et al. [93] found a total of 26 upregulated and 17 downregulated miRNAs following in vitro decidualization of hESC. Interestingly, only miR-155 was commonly downregulated in both studies, and no change was found in the expression of miR-542-3p in the study of Estrella et al. Despite several differences and significant variability, probably attributable to decidualization in vitro, these studies have nonetheless shown that the miRNA profiles of the proliferative versus secretory endometrium are significantly different, and therefore deserving of further research.

### Endometrial fluid

Endometrial fluid is the medium where the most part of the exchanges between the embryo and the endometrium occur, allowing for a tight bidirectional regulation [94–96]. Among the substances conveyed in the endometrial fluid, extracellular vesicles (EVs) are emerging as one of the most important mediators, particularly in relation to their capability of transporting ncRNAs [2, 97–99]. The secretion of exosomes from the apical surface of endometrial glands, which was confirmed by electron microscopy, is consistent with the existence of endosomes in epithelial cells and of embryo-derived extracellular vesicles [100]. Furthermore, primary human endometrial endothelial cells (hEECs) were found to actively secrete large quantities of exosomes into the conditioned media, and labelling experiments with miR30-d revealed that miRNAs are internalized in vesicles and secreted in exosomes. Several experiments showed that exosomes loaded with miR-30d were secreted from hEECs and could be internalized by trophoblastic cells of murine embryos adhered to hEECs [96]. Also, hEECs have been demonstrated to be able to uptake embryo-derived miRNAs, such as the embryo–endometrial adhesion inhibitor miR-661 [101]. Recent literature revealed the presence, in the endometrial fluid of both fertile and infertile women, of 12 sncRNAs strongly associated with biological functions related to immune response, extracellular matrix and cell junction, highlighting the different expression patterns in the two subpopulations and suggesting that sncRNA could be used as biomarkers of endometrial receptivity and implantation success [102].

Even though the capability of numerous noncoding RNAs (both maternal and embryo-derived) of being transmitted through endometrial fluid has been clearly established, the role of such molecules in the regulation of pivotal phases of implantation and early embryonic development still requires much needed investigation [98].

## Hot spots of epigenetic action at the fetal stage

Further processes of vascular remodeling, trophoblastic cellular migration, and immune regulation allow the formation of the placenta, a transient organ that connects the fetus to the mother [103]. The placenta supports fetus development supplying oxygen and nutrition, protects the semi-allogeneic fetus from immune rejection, and secretes hormones that affect maternal organs in order to promote the maintenance of pregnancy [104–107]. Many maternal influences, such as over and under-nutrition, drug and alcohol intake, smoking, infection, stress, and hormones activity (for example glucocorticoids [GCs] or thyroid hormones), can induce transformations in placental physiology. These placental modifications can range from alterations in aspects of macroscopic placental morphology to more subtle changes in placental gene expression which may have long-term effect on offspring health. However, many mechanisms of transitory prenatal insults that result in postnatal dysregulation remain unclear [108].

A pivotal role in the fetal stage of pregnancy is played by the amniochorionic membranes, which act as the feto-maternal interface, as they exhibit characteristic chromatin modification patterns, DNA (CpG) methylation, histone modifications, and non-coding RNA transcriptomes, whose dynamic changes during normal and pathological pregnancies are an important contributor to gene regulation throughout pregnancy [31]. Alterations in DNA methylation patterns and ncRNAs in amniochorionic membranes have also been detected in association with preterm birth and pathological conditions such as acute chorioamnionitis [109].

Numerous miRNAs are predominantly or exclusively expressed by the placenta and can be found clustered in specific chromosomal regions; they may be also controlled by the same promoters, have similar seed regions and targets, and work synergistically. Placental miRNome studies have also demonstrated its importance towards the coordination and modulation of the placental transcriptome [110, 111]. The three most relevant clusters are the chromosome 19 miRNA cluster (C19MC), the chromosome 14 miRNA cluster (C14MC), and the miR-371–3 cluster, which is localized on chromosome 19 as well. These miRNAs primarily regulate placental processes such as the migration of the trophoblast and the placental morphogenesis, but evidence has emerged that they are transported to the fetal and maternal compartments as well, even though their role in such districts is still unclear [80, 82, 83].

Epigenetic processes within the placenta are powerful mediators of maternal and environmental signals to the developing fetus. Furthermore, alterations of placental gene expression and signaling during fetal development can dramatically change the developmental program, particularly concerning brain development [110].

### Placental metabolism and brain development

Among the organogenetic processes that occur during fetal development, one of the most critically affected by maternal, environmental, and epigenetic factors is brain development. The initial steps of nervous system development start as early as 2–3 weeks post fertilization in humans; however, neuronal proliferation begins later in the first trimester and synaptogenesis and neural migration mainly occur in the later stages of pregnancy, during the second and third trimesters. In the early pregnancy, the brain is extraordinarily plastic, but also exposed to environmental fluctuations which can influence long-term programming. The developing brain tissue requires considerable nutritional intake and it is particularly susceptible to overabundance or insufficiency of specific nutrients and growth factors [108, 110, 111].

Both maternal and fetal factors contribute to neurodevelopment during gestation [112], and the placenta is specifically believed to play a pivotal role [108, 113, 114].

The brain is an organ that consumes a lot of energy and absorbs a massive quantity of maternal resources during its growth and evolution. It is also specifically vulnerable to modifications in placental activity. Many signals regulate chromatin activation or repression; among these, those related to nutrient availability are relevant for epigenetic programming in the placenta.

In this scenario, the X-linked enzyme O-linked-N-acetylglucosamine transferase (OGT) seems to have a fundamental role in neurodevelopmental organization [108, 110, 115–118]. In fact, OGT represents a link between nutritional signals and chromatin regulation. It works as a nutrient sensor which modifies various proteins in order to change numerous cellular processes, like major epigenetic changes (such as methylation) [110].

The signaling of insulin, IGF-1 (insulin-like growth factor-1), and leptin receptors located at the maternal–fetal interface promotes amino acid transporter activity in trophoblast cells, combining maternal nutritional state to placenta function and, consequently, invalidating the accessibility of nutrients diffusing into the fetal circulation [119].

Some authors have reported that increased methylation of the leptin receptor gene in the human placenta is associated with increased lethargy and hypotonicity in male but not in female newborns [120]. This hypothesis supports both a connection between epigenetic regulation of the leptin receptor and the communication of fundamental information to the fetal brain and also a certain sex-specificity in these results.

To date, the relationship between placental metabolism and epigenetic programming has been especially studied in animals (and only marginally in humans) with particular attention to sex development, as sex differences in epigenetic mechanisms such as DNA methylation have been described across placental development. In mice, female placental tissue has higher amounts of global DNA methylation compared to male placentas, which may confer females additional protection from constant alterations in gene expression due to environmental insults [108, 120–122].

Therefore, the recognition of relevant placental biomarkers, such as OGT and leptin, that, in response to maternal nutrition and stress hormones levels, produce epigenetic modifications which sensibly affect the neurodevelopmental programming, may allow in the future for a better understanding and prevention of neurodevelopmental disorders in the offspring [123].

### Placental neurotransmitters and neurobehavioral outcomes

The placenta is known to be involved in the transfer of nutrients, hormones, and metabolites, but it is important to remember that it also produces neurotransmitters, including serotonin (5-HT), dopamine (DA), norepinephrine/epinephrine, which enter the maternal–fetal circulation and interact with the development of the fetal brain. Therefore, it is reasonable to assume that some neurobehavioral disorders (such as autism spectrum disorders, ASD) may originate from placental changes in the production of these metabolites. For instance, 5-HT promotes various processes during fetal brain growth: neuronal migration, cell division, and differentiation and synaptogenesis. While placental hyperserotonaemia can disrupt early neural development, hyposerotonaemia too appears to impair cognitive, motor, or sensory capacities [118, 124].

Recently, DNA methylation and miRNA expression patterns within the placenta have been also studied for potential associations with later neurobehavioral disruptions in the offspring [118]. Although the exact mechanisms are still unclear, here, we summarize the literature about the connection between epigenetic modifications and neurobehavioral disorders.

Some authors reported, in a prospective study of high-risk pregnancies, that 400 DMRs discriminate placentas from offspring later diagnosed with ASD compared to those not diagnosed with such disorders [124].

Besides placental 5-HT affecting neurobehavioral development, methylation of placental HTR2A (the receptor which mediates the effects of 5-HT) may also be implicated. HTR2A methylation correlates inversely with infant quality of movement, but positively with infant attention [125].

Altered methylation patterns of 10 imprinted genes (DLX5, DHCR24, VTRNA2-1, PHLDA2, NPA1, FAM50B, GNAS-AS1, PAX8-AS1, SHANK2, and COPG2IT1) have been also associated with reduced quality of movement, elevated indices of asymmetrical and non-optimal reflexes, and increased likelihood of physiological stress [126].

Another type of epigenetic modification that can occur in the placenta and may regulate infant neurobehavioral patterns is represented by the alterations in miRNA expression profiles. Increased placental miR-16 expression is negatively associated with attention score. In contrast, miR-146a and miR-182 are positively related to quality of movement score [118]. In conclusion, even if the placenta is a temporary organ, there is no doubt that its alterations can strongly influence the development of the fetus, and in particular of the fetal brain. Biomedical research is currently focused on identifying placental biomarkers useful for identifying fetuses at risk of developing neurodevelopmental disorders, for starting an early clinical management [118, 125–129].

## Role of maternal and environmental factors during pregnancy

### Stress hormones

Several data suggest a direct association between prenatal early-life stress (i.e., maternal depression, chronic stress, abuse) and the incidence of psychopathology and cognitive impairments in the baby. Early life stress acts in different ways in different tissues, altering gene expression and inducing epigenetic modifications (e.g., increased or reduced DNA methylation or histone acetylation in many brain regions at the same time) [96].

In animals, stress during pregnancy and increased glucocorticoids (GCs) levels cause alteration in stress hormones receptors and impair the feedback regulation of the hypothalamic-pituitary adrenal axis in infancy and adulthood. Stress acts also on the amygdala and increases the incidence of anxiogenic and depressive-like behaviors [130].

In humans, excess amounts of corticotropin releasing hormones (CRH) and cortisol reaching the fetal brain can alter personality and predispose to attention deficits and depressive illness through changes in neurotransmitter activity [9, 73]. In fact, neurobehavioral alterations have been related to pre-gestational stress. In vitro studies showed that stress hormones can increase excitability hippocampal cells [131].

However, a recent review concluded that most of the published papers on this topic did not reveal a significant association between maternal cortisol in pregnancy and poorer offspring birth outcomes, lower cognitive outcomes, lower cognitive/motor development, or more behavioral problems in infancy and childhood. This result probably reflects the numerous confounding factors that act synergistically during pregnancy and in the postnatal period [132].

### Nutrition and environment

The first 1000 days, which start from pre-conception until approximately two years of age, are considered the period during which early nutrition, thanks to its influence on epigenetic changes [133], can play a key role in developmental programming and can influence the individual susceptibility to diseases later in the life (cardiovascular diseases, obesity, diabetes, and other chronic condition). Patients with eating disorders show adverse pregnancy outcomes such as miscarriage, preterm delivery, or fetal anomalies as poor fetal growth or malformations. Altered levels of many nutrients (i.e., vitamin B, folic acid, zinc) can increase the risk of developing fetal disorders [134, 135].

Early life nutrition can modulate the epigenome through different mechanisms: the change in the structure of chromatin through histone modifications and the supply of methyl donors (i.e., methionine, choline, folate), the activity of DNA methyltransferases, and of specific transcription factors. In this way, altered maternal nutrition may induce epigenetic changes in the global expression pattern of the fetus, which will trigger biological and psychological alterations in offspring’s lifelong outcomes [136–139].

Moreover, some nutrients, such as vitamin B and polyphenols, can modulate the function of the methylation enzymes [140]. In particular, Vitamin B12 is associated with one carbon metabolic pathway and to substrate metabolism, as well as to the synthesis and stability of nucleic acids and methylation of DNA [137, 141]. High levels of vitamin B12 in the maternal blood was correlated with the reduction in the total level of DNA methylation of the neonate, while the elevated concentration of serum vitamin B12 in the new-born correlated with decrease methylation levels of the insulin-like growth factor-binding protein3 (IGFBP-3) gene, which is one of the candidate genes for intrauterine growth [142].

The lack of nutrients during pregnancy has been associated with various later-life consequences. Famine during fetal life, for example, can increase the risk of developing glucose intolerance or coronary artery disease in adulthood, creating persistent changes in DNA methylation that depend on the sex of the exposed individual and the gestational timing of the exposure [143, 144]. On the other hand, maternal obesity and a high fat diet can create not only metabolic disorders but also neurodevelopmental morbidity in the offspring. Maternal overeating and low protein consumption during pregnancy is associated with significant miRNA dysregulation in the offspring tissue, which may be associated with chronic inflammation status and metabolic health in offspring as early as the weaning age [145, 146]. As suggested in the recent literature, modifications to the offspring’s epigenome could even be influenced by paternal nutrition, mediated by epigenetic modifications in sperm [147].

One of the most common pregnancy complications worldwide correlated to nutrition is gestational diabetes (GDM). Unhealthy nutritional habits and excessive gestational weight gain during pregnancy can predispose to maternal GDM [148, 149].

GDM increases the incidence of short-term (i.e., large for gestational age, shoulder dystocia, higher body fat) and long-term complications (i.e., obesity, metabolic syndrome, diabetes mellitus type 2, attention problems, and depression) observed in the offspring [150, 151]. These effects are regulated by alteration in the epigenetic programming. Maternal GDM can create different effects like global DNA hypermethylation or alteration of miRNA expressions. A recent study shows that the effect of diabetic environment on miRNA regulated endothelial dysfunction is sex dependent, as females are more susceptible to metabolic derangements of GDM than males [151, 152].

GDM changes also placental microbiota: for example, in women with GDM, there are less bacteria belonging to the Pseudomonadales order and Acinetobacter genus associated with a more adverse metabolic and inflammatory phenotype. In this way, GDM could represent a state of placental microbiota-driven altered immunologic tolerance that can be the target of a new therapy [153].

Interestingly, epigenetic markers of the GDM offspring could become a tool for early detection and prognosis of adverse phenotypic outcomes [154].

Beyond food, there are many substances which influence epigenetic programming. Endocrine disruptors (EDs), for example, are environmental pollutants that mimic endogenous hormonal signals. The EDs most commonly associated with reproductive abnormalities are the xenoestrogens such as Bisphenol-A (BPA), polychlorinated biphenyls (PCBs), and antiandrogens such as phthalates. These compounds exhibit weak steroid-like activity and therefore can affect reproductive development along multiple points including the hypothalamus and the gonad. Exposure to EDs starts during fetal life and continues after birth: the link between prenatal exposures and latent health outcomes suggests that these exposures may result in long-term epigenetic reprogramming.

The epigenomic changes, due to prenatal exposure to ED, include altered global DNA methylation, gene specific CpG methylation, and microRNA expression [155]. In human cell lines, PCB exposure has been shown to modulate the activity of histone demethylases via androgen receptor binding [156]. Moreover, other EDs have been shown to interact with DNMTs and TETs enzymes, altering their activity in in vitro experiments [157]. Additionally, some EDs are known to interact with one-carbon metabolism, which produces methyl donors used for DNA methylation [158]. Finally, it is probable that EDs alter the transcription factors’ activity, changing the availability of DNA to epigenetic machinery and giving rise to gene-specific patterning of epigenomic markers [159]. Therefore, these epigenetic changes represent a critical mechanism essential to understand the changes of fetal epigenome causing adverse outcomes at birth and later in life, warranting further study.

### Teratogens

In addition to stress, nutrients, and endocrine disruptors, other environmental factors, such as medications, alcohol or substances of abuse, produce changes in the global pattern of gene expression as well as hormonal imbalance, and ultimately adverse effects on embryonic development.

One of the best known and most studied teratogens is ethanol, for it can cause a wide range of developmental abnormalities. Alcohol-related neonatal abnormalities are commonly referred to as fetal alcohol spectrum disorder (FASD) [160]. The most severe form of FASD is called fetal alcohol syndrome (FAS) and manifests as growth retardation, facial abnormalities, and central nervous system deficiencies. Even if there is no specific time point during gestation when alcohol exposure is not accompanied by harmful consequences, the exposure during early embryonic stage is correlated with most severe birth defects [161]. Many researches have also found that alcohol interferes with gene expression levels and epigenetic processes by altering DNA methylation (particularly the expression of two methyltransferase enzymes, DNMT1 and DNMT3A), histone regulation, and non-coding RNAs [162]. Alcohol also induces alterations in the DNA methylome of the hypothalamus, including several differentially methylated regions (DMRs) that could underlie some of the deficits observed in FASD. DNA methylation profiles may not persist into adulthood but could alter developmental trajectories and induce lasting alterations in brain structure, connectivity, and function [163].

Children prenatally exposed to moderate-high levels of alcohol showed increased DNA methylation of stress regulatory genes proopiomelanocortin (POMC) and period 2 (PER2) resulting in increased levels of stress hormone cortisol and adrenocorticotropic hormone (ACTH), compared to controls. These results suggest the possibility that measuring DNA methylation levels of PER2 and POMC in biological samples from pregnant women or from children can serve as a biomarker of alcohol-related disorders [164].

Further studies are definitely needed to understand the correlation in detail between fetal alcohol spectrum disorder and epigenetic changes [165].

Unfortunately, smoking during pregnancy is still very widespread, especially in some populations where the level of attention to this teratogen is not so high. Maternal tobacco smoking is associated with smaller birth weight and head circumference, as well as reduced length for gestational age [166, 167]. Moreover, maternal tobacco smoking is associated with an increased risk of obesity [168], respiratory infection [169], and cardiovascular [170], psychiatric, and behavioral disorders [171–173].

In addition to maternal smoking, exposure to air particulate matter, polycyclic aromatic hydrocarbons, arsenic, heavy metals, cannabinoids, and persistent organic pollutants during pregnancy has been associated with epigenetic changes [174, 175]. Cigarette smoke creates alterations in DNA methylation of cord blood and placenta, histone modifications, and miRNA expression. In utero tobacco exposure, even in the absence of fetal growth restriction, may alter the epigenome, contributing to global DNA hypomethylation. Investigation towards miRNA and downstream transcriptional regulation are growing, while studies investigating histone modifications are still sparse [176].

Even second-hand smoke exposure among non-smoking women may alter DNA methylation in regions involved in development, carcinogenesis, and neuronal functioning. These novel findings suggest that even low levels of smoke exposure during pregnancy may be sufficient to alter DNA methylation of the fetus [177]. Hopefully, in the future, DNA methylation status could be used as a biomarker of prenatal insults for tobacco exposure as well.

Regarding substances universally recognized as teratogens, one of the most widespread is valproic acid (VPA). VPA is a well-tolerated antiepileptic drug and mood stabilizer that is used for the treatment of epilepsy and other non-psychiatric diseases like Alzheimer, HIV, and cancer [178]. VPA interacts with the inhibitory neurotransmitter gamma-Aminobutyric acid (GABA) and blocks voltage-gated ion channels. Several authors demonstrated on murine models that early increases in histone acetylation occur within the embryo and in murine decidua following gestational VPA exposure [179, 180]. This mechanism of action is known to result in teratogenicity and cell toxicity, causing a wide range of fetal alterations, both malformative and neurological.

Some other commonly used drugs have specific fetal body effects: a recent study, for example, has demonstrated that cyclophosphamide induces limb dysmorphogenesis by alterations in some miRNA expression (i.e., miRNA-34, miRNA-125b, and miRNA-155). These miRNAs, in fact, act in different ways: some miRNAs act to protect embryos, whereas other miRNAs boost a teratogen-induced process of maldevelopment to induce embryonic death. The analysis of correlations between the expression pattern of the miRNAs tested in the study and cyclophosphamide induced limb phenotypes implies that miRNAs regulating apoptosis may differ from each other with respect to their functional role in teratogenesis [181].

Finally, regarding another known teratogen inducing limb reduction anomalies (5-aza-2’-deoxycytidin or 5-aza), recent animal study focused the attention on miR-34 family, concluding that 5-aza and cyclophosphamide are able to activate miR-34 family in p53-independent fashion. This observation implies a scenario in which miR-34 family plays a regulatory role in the response to embryopathic stresses not activating the p53 pathway, but more studies are needed to explore these mechanisms. It is also undoubtedly important to reveal whether other teratogens can affect offspring in this way [182].

## Conclusions

The knowledge regarding the feto-maternal cross talk is rapidly expanding and an ever-increasing amount of evidence has been highlighting the importance of epigenetic regulation both in determining the effectiveness of early impregnation processes and in influencing fetal development. While many of the epigenetic mechanisms affecting early and late embryo development have long been known and studied, a promising new frontier comprising ncRNAs and neurodevelopmental factors has yet to be fully explored and understood, especially regarding the human species. Determining the precise mechanisms underlying such phenomena could prove extremely valuable towards a better management of both physiological pregnancies and assisted reproduction treatments. A better understanding of the role of epigenetic agents in embryonic development may ultimately improve counselling in infertile couples, provide new pharmacological treatments to favor the early phases of the pregnancy, and allow a better recognition and management of potentially harmful or even beneficial environmental factors with regards to fetal development.
